# Assessing the Quality of Urological Operation Notes in Accordance With the Royal College of Surgeons of England Guidelines: A Retrospective Cross-Sectional Study

**DOI:** 10.7759/cureus.71845

**Published:** 2024-10-19

**Authors:** Mohammed Elsiddig, Mohammed Hassan

**Affiliations:** 1 Urology, Military Hospital, Khartoum, SDN

**Keywords:** operative notes, quality improvement projects, rcs guidlines, safety patient, surgical practice

## Abstract

Objective

This study aimed to assess the practices of documenting operative notes in the urology department at a tertiary care facility in Sudan.

Materials and methods

This cross-sectional, retrospective study evaluated the practices of documenting surgical notes for patients who required urological procedures at a tertiary hospital in Sudan.

The study included all patients who underwent emergency or elective urological surgery between March 1, 2023, and April 1, 2023, under general or spinal anesthesia, regardless of age.

The medical records were accessed to analyze the operative notes' standards. Sixty-two operative notes were included and analyzed. We used the Royal College of Surgeons of England (RCS) guidance as the standard for our study. The Royal College of Surgeons of England has provided comprehensive and standardized guidelines for writing an operative note, including patient identification, date and time of the procedure, type of procedure (elective/emergency procedure), name of surgeon and assistant, name of anesthetist, name of the procedure, type of incision, operative diagnosis, operative findings, complications encountered, any extra procedure performed with reason, details of tissue removed, added or altered details of closure technique, anticipated blood loss, prosthesis details, antibiotic prophylaxis, deep vein thrombosis prophylaxis, detailed post-op instructions, signature.

Results

A total of 62 consent forms were included. Patient identification, name of the surgeon and assistant, and name of the procedure were mentioned in 61 (98.4%). The operative diagnosis and details of tissue removed were written in 41 (66%). Antibiotic prophylaxis and deep vein thrombosis prophylaxis were given in 28 (45.2%) and 25 (40%), respectively. The type of anesthesia was mentioned in 56 (90.3%) of cases. The name of the anesthetist and anticipated blood loss: recorded completion rates of 12 (19.3%) and 18 (29%), respectively. The date and time of the procedure were completely documented in 59 (95.2%).

Senior doctors wrote six (9.7%) of the operative notes with a completeness of four (66.7%). Junior doctors wrote 56 (90.3%) operative notes, and only 12 (21.4%) completed the essential components of the notes.

Conclusions

The current practices of documenting operating notes were shown to be imprecise. Handwritten operative notes do not follow standard practice. Using a pre-designed operating note that may be customized according to the circumstances of the operation is the preferred option. An educational session should be planned for junior doctors to focus on correct documentation and promote adherence to best clinical practices.

## Introduction

Surgical operation notes serve as a means of communication among the many members of the interdisciplinary healthcare team who are involved in the process of planning and carrying out a surgical procedure [1]. To ensure safe patient care and follow-up, surgeons must meticulously document each surgery in their operating notes [1]. These notes also have use in quality assurance, teaching, medicolegal matters, and research [2,3]. Operation notes must be accurate and easy to read to ensure the safe continuation of treatment for surgical patients.

The 2014 publication of "Good Surgical Practice" by the Royal College of Surgeons of England (RCS) laid forth their standards, which have since become the norm for operating room notes. Providing clarity and conciseness that is relevant across different surgical departments, these recommendations provide eighteen fundamental requirements for the production of operational notes. Conformity with these standards demonstrates a determination to improve the standard of surgical documentation, which in turn supports the larger goals of good clinical governance [4].

A significant stride toward perfect record-keeping has been the implementation of standardized proforma for operating notes. There are difficulties concerning readability because most operating notes are written by hand in many places of the world [5]. Typing notes has shown promise for improving documentation quality and decreasing mistakes. Improved readability, better time management, and smoother communication between different sections of the healthcare institution are just a few of the many benefits of electronic notes over handwritten ones [6,7].

The operating note is traditionally handwritten at our institution, including sections allowing the surgeon to specifically describe the operation. In this context, the operative note may not be completely filled out. This study set out to evaluate the adherence to RCS guidelines in the writing of operating notes for urological operations at a tertiary teaching hospital in Sudan.

## Materials and methods

This cross-sectional, retrospective study evaluated the practices of documenting surgical notes for patients who required urological procedures at the Military Hospital, Khartoum, a tertiary hospital in Sudan, between March 1, 2023, and April 1, 2023.

For the purpose of evaluating the quality of the surgical notes, the medical records were accessed. The study included all patients who underwent emergency or elective urological surgeries between March 1, 2023, and April 1, 2023, under general or spinal anesthesia, regardless of age. No notes were included for operations that required local anesthesia or for diagnostic procedures. Two authors reviewed the operating notes for legibility. 

Sixty-two operative notes were included and analyzed. We used the Royal College of Surgeons of England (RCS) guidance as the standard for our study. The Royal College of Surgeons of England has provided comprehensive and standardized guidelines for writing an operative note, including patient identification, date and time of procedure, type of procedure (elective/emergency procedure), name of surgeon and assistant, name of anesthetist, name of procedure, type of incision, operative diagnosis, operative findings, complications encountered, any extra procedure performed with reason, details of tissue removed, added or altered details of closure technique, anticipated blood loss, prosthesis details, antibiotic prophylaxis, deep vein thrombosis (DVT) prophylaxis, detailed post-op instructions, and signature.

A Google Form (Google Inc., Mountainview, CA) was created and included all the necessary components of the operative note. To facilitate straightforward analysis, each section required a simple "yes" or "no" answer (Appendix 1). This study used IBM SPSS Statistics for Windows, Version 22 (Released 2013; IBM Corp., Armonk, New York, United States) to tabulate and analyze the data. We utilized measures such as central tendency, percentage, and frequency to assess the categorical data. The chi-square test was used to evaluate the relationship between the level of the doctor and the completeness of operative notes. The significance level was established at P < 0.05.

Obtaining ethical approval was not required as the study was carried out inside the urology department and used a retrospective analysis of existing documents.

## Results

An evaluation of 76 operative notes was conducted, and 62 notes were included in the study. Patient identification, name of the surgeon and assistant, and name of the procedure were mentioned in 61 (98.4%). The operative diagnosis and details of tissue removed were written in 41 (66%). Antibiotic prophylaxis and DVT prophylaxis were present in 28 (45.2%) and 25 (40%), respectively. The type of anesthesia was recorded in 56 (90.3%) of cases. The name of the anesthetist and anticipated blood loss: recorded completion rates of 12 (19.3%) and 18 (29%), respectively. The date and time of the procedure were completely documented in 59 (95.2%). The completeness of the essential components of the operative notes is shown in Table 1.

**Table 1:** The completeness of the essential components of the operative notes Data are presented as N (%) for the presence and absence of each component. DVT: deep vein thrombosis

Component assessed	Present N (%)	Absent N (%)
Patient identification	61 (98.4%)	2 (3.2%)
Date and time of procedure	59 (95.2%)	3 (4.8%)
Type of procedure (elective/emergency procedure)	45 (72.6 %)	17 (27.4%)
Name of surgeon and assistant	61 (98.4%)	1 (1.6%)
Name of anesthetist	12 (19.3%)	40 (80.7%)
Name of procedure	61 (98.4%)	1 (1.6%)
Type of anesthesia	56 (90.3%)	6 (9.7%)
Type of incision	42 (68%)	20 (32%)
Operative diagnosis	41 (66%)	23 (34%)
Operative findings	56 (90.3%)	6 (9.7%)
Complications encountered	31 (50%)	31 (50%)
Any extra procedure performed with reason	25 (40%)	37 (60%)
Details of tissue removed, added, or altered	41 (66%)	23 (34%)
Details of closure technique	45 (72.6 %)	17 (27.4%)
Anticipated blood loss	18 (29%)	44 (61%)
Prosthesis details	49 (79%)	13 (21%)
Antibiotic prophylaxis	28 (45.2%)	34 (54.8%)
DVT prophylaxis	25 (40%)	37 (60%)
Detailed post-op instructions	55 (88.7%)	7 (11.3%)
Signature	52 (83.9%)	10 (16.1%)

Senior doctors wrote six (9.7%) of the operative notes with a completeness of four (66.7%). Junior doctors wrote 56 (90.3%) operative notes, and only 12 (21.4%) completed the essential components of the notes. The correlation between the completeness of the essential components of the operative notes and the levels of the surgeon is shown in Table 2.

**Table 2:** The level of the doctor who wrote the operative notes and completeness of the notes Data are presented as N (%) for operative notes and completeness.

Level of doctor	Number (%) of operative notes	Number (%) completeness of the operative notes
Consultant	6 (9.7%)	4 (66.7%)
Trainee	56 (90.3%)	12 (21.4%)

We used the chi-square test to look at how the number of operating notes varied according to the doctor's level (consultant vs. trainee). Consultants' and trainees' note-taking rates of completeness were significantly different, according to the data. Consultants and trainees differ significantly in the extent to which their operating notes are comprehensive, as shown by the p-value of 0.022 (p < 0.05). The result of the chi-square test evaluating the completeness of operative notes by the level of doctor is shown in Table 3.

**Table 3:** Results of the chi-square test evaluating the completeness of operative notes by the level of doctor The chi-square statistic of 5.23 indicates the degree of deviation from the expected frequencies under the null hypothesis. Degrees of freedom (1) and p-value (0.022) are provided, indicating a significant difference (p < 0.05) in documentation quality between consultants and trainees.

Statistic	Value
Chi-square statistic (χ2\chi^2χ2)	5.23
Degrees of freedom (df)	1
p-value	0.022
Significance level	p < 0.05
Conclusion	A significant difference in the completeness of operative notes between consultants and trainees

## Discussion

Operational notes are among the most crucial medical documents. The entire surgical team may simplify their patient management strategy with a thorough record. Proper documentation of a surgical operation is essential for postoperative patient care, and one of the most significant abilities a surgeon must possess is the ability to write accurate and comprehensive operative notes. This is seen as a fundamental component of surgical quality assurance [8].

Postoperative patient care is impacted by incomplete operation notes. For instance, notes with illegible handwriting or nonstandard abbreviations might confuse healthcare workers responsible for the patient's continued treatment.

A previous study found that as many as 45 percent of operation notes could not be utilized to support a defendant in a court of law, highlighting the inutility of inadequate notes for non-medico-legal matters [9].

According to our study, the level of expertise and education of the operating doctor can affect the accuracy of surgical notes. Within this study, a total of 56 (90.3%) notes were written by junior doctors, with only 12 (21.4%) completing the essential parts of the operating notes. Conversely, the consultant wrote six (9.7%) of the operative notes, with four (66.7%) of those notes meeting all the necessary criteria. The results show a strong relationship between the number of doctors who got the consent and how thorough it was, which is in line with other research [10]. It would be helpful to provide junior doctors with structured training focusing on high-quality operative documentation and equip them with the tools required to adhere to established guidelines for postoperative notes, which will ultimately enhance the quality of healthcare delivery [8,11].

Consistent with another study, 56 (90.3%) of the surgical notes we reviewed included information about the anesthetic and intraoperative results [12]. Compared to the rates shown in the aforementioned study carried out in Nigeria, our study showed lower rates in the documentation of the surgical diagnosis, complications encountered, and closure method [13].

Findings from this study are consistent with those from other studies in Africa and abroad on the documentation of the surgery date, which was mentioned in 95% of the notes [8,14]. While the names of the surgical team members were recorded in the notes rather consistently in this research, the name of the anesthetist was mentioned in 19%. This finding is in line with prior research conducted in Sudan, which also discovered that the names of the anesthetists were very seldom recorded [14].

There has to be an improvement in the quality of documentation of patients' perioperative data because no operation note was filled out adequately in this study. The findings of audits conducted in Sudan and other African countries have been comparable, and in some cases even worse [14,15].

Recommendations

We recommend that hospital administrators apply standardized operation notes that adhere to the RCSE guidelines as a starting point for improving and standardizing note-writing. These notes should include clear and itemized sections for the following: the patient's preoperative condition, the urgency of the procedure, the members of the surgical team, the date and time of the procedure, details about the intraoperative procedure, and postoperative care instructions.

We advise organizing teaching sessions and periodic training of junior doctors focused on proper documentation to promote adherence to guidelines. 

Limitations

The limited sample size is the main constraint of our study since it might affect the reliability and statistical strength of our results. Another limitation is that the study only included one facility, which would restrict how generalizable the findings are.

## Conclusions

The current practices of documenting operating notes were shown to be imprecise. Handwritten operative notes do not follow standard practice. Using a pre-designed operating note that may be customized according to the circumstances of the operation is the preferred option. An educational session should be planned for junior doctors to focus on correct documentation and promote adherence to best clinical practices.

## Appendices

Appendix 1 

**Table 4:** Questionnaire for assessing the quality of urological operation notes Please select Yes or No for each component to indicate whether the operative note contains the relevant information DVT: deep vein thrombosis

Component	Yes	No
Patient identification		
Date and time of the procedure		
Type of procedure		
Name of surgeon and assistant		
Name of anesthetist		
Name of procedure		
Type of anesthesia		
Type of incision		
Operative diagnosis		
Operative findings		
Complications		
Any extra procedure performed with reason		
Details of tissue removed, added, or altered		
Details of closure technique		
Anticipated blood loss		
Prosthesis details		
Antibiotic prophylaxis		
DVT prophylaxis		
Detailed post-op instructions		
Signature		
Level of Doctor		
Consultant		
Trainee		
